# Parenting stress and needs for social support in mothers and fathers of deaf or hard of hearing children

**DOI:** 10.3389/fpsyg.2023.1229420

**Published:** 2023-08-30

**Authors:** Amélie Marie, Laëtitia Clabaut, Marjolaine Corbeil, Clémence Vanlerberghe, Catherine Vincent-Delorme, Barbara Le Driant

**Affiliations:** ^1^CRP-CPO (UR 7273), University of Picardie Jules Verne, Amiens, France; ^2^Reference Center for Rare Diseases « Genetic Deafness », CHRU, Lille, France

**Keywords:** parenting stress, social support, deaf or hard of hearing, mothers-fathers differences, needs

## Abstract

**Introduction:**

Hearing parents of deaf or hard of hearing (DHH) children may experience parenting stress and social support could be a buffer to this stress. Differences in levels of these two indicators may exist between mothers and fathers. This study focuses on the parenting stress and social support needs of mothers and fathers of DHH children.

**Methods:**

Twenty-seven French parental couples of DHH children completed the Parenting Stress Index and the Family Needs Survey, a questionnaire on social support needs.

**Results:**

Their overall stress scores showed no difference, but subdomain scores show that mothers and fathers are more stressed by the child’s hyperactivity, and fathers by the child’s adaptability, than parents of children with normal hearing. Mothers are more stressed than fathers by role restriction; they feel less free because of their parenting role. Fathers have a lower quality of attachment to their child than mothers. Parents have a high social support need, especially for obtaining information about their child’s individual characteristics and health situation. The ranking of mothers and fathers in the top 10 needs reveals different needs profiles. Parenting stress profiles show that mothers and fathers with higher-than-normal stress levels have a greater overall need for social support than mothers and fathers with lower than normal stress levels.

**Discussion:**

This study highlights the value of assessing parenting stress and social support needs in parents of DHH children for a better understanding of their situation in research and its clinical implications, as well as the importance of differentiating outcomes for mothers and fathers.

## 1. Introduction

### 1.1. Parenting stress

Parenting stress is a psychological state of tension in response to the demands of raising a child. Both child and parent factors can generate parental stress (Abidin, 1983). The announcement of a diagnosis of deafness in a child is a major source of stress for parents who, in 90% of cases, are hearing and inexperienced in deafness matters (Mitchell and Karchmer, 2004). Hearing parents of deaf or hard of hearing (DHH) children have to face multiple challenges. In particular, they will have to make choices of communication mode (oral vs. signed), rehabilitation process, and early intervention programs (Fitzpatrick et al., 2008). The lack of experience of hearing parents of DHH children and the concerns that may result can lead to more stressful parenting (Kurtzer-White and Luterman, 2003). In the field of deaf studies, research on parenting stress in hearing parents of DHH children has produced a wide range of findings without reaching a consensus. Both parents of DHH children did not have higher level of distress compared to parents of children with normal hearing, but 20% reported depression, anxiety and moderate to extremely severe stress (Kasin et al., 2020). Other research has not found the same correlation between parenting stress and raising a DHH child. For example, Brand and Coetzer (1994) found no difference in stress levels in mothers and fathers, but at higher socio-economic levels, they reported lower stress levels. More recently, the same finding has been made and the comparison of parents of DHH children and parents of children with normal hearing found no difference in stress (Åsberg et al., 2008; Chang et al., 2022). Studies frequently consider parents as a unit, without differentiating mothers and fathers. While it is true that mothers and fathers of children with disabilities can express similar needs or experiences in parenting (Cardinali et al., 2019), the behaviors and experiences of mothers and fathers of DHH children could not be seen as interchangeable (Zaidman-Zait et al., 2017), especially as the role of fathers has evolved in the last few decades, leading them to become more involved in the care and education of their children. For example, in a longitudinal study, mothers of DHH children at 22 months old expressed higher stress in response to child characteristics but at 4 years old no greater stress was found. However, this study did not use the same test at both ages (Lederberg and Goldbach, 2002). Another study showed that mothers of DHH children at 4 years old expressed more stress than mothers of children with normal hearing (Quittner et al., 1990, 1991). A more recent study investigated paternal stress: fathers of 4.5-year-old DHH children experienced more stress than fathers of children with normal hearing (Mavrogianni and Lampropoulou, 2020). Some variables seem to influence parenting stress. Mothers and fathers of DHH children with cochlear implant register more stress than those of children with hearing aids: the rehabilitation with cochlear implant being more stressful, especially in the early stages (Spahn et al., 2003; Burger et al., 2005). The degree of hearing loss could influence parenting stress: mothers of DHH children with milder hearing loss have been found to be more stressed than mothers of children with more severe hearing loss (Pipp-Siegel et al., 2002), though Zaidman-Zait et al. (2016) did not find any relationship between degree of hearing loss and parenting stress.

Abidin (1990) noted early on that his model of parenting stress could benefit from considering other factors, as factors not included in this model, such as social support, may influence the stress experienced by parents.

### 1.2. Social support and parenting stress

In the case of parents of DHH children, Meadow Orlans has postulated since 1994 that social support acts as a buffer to stress. Indeed, mothers who perceive more social support have lower levels of stress and the same correlation seems to be present in fathers. More recent surveys showed a similar relationship, finding an association of social support and parenting stress in mothers and fathers of DHH children. When they experienced higher levels of social support, they showed lower levels of parenting stress (Pipp-Siegel et al., 2002; Åsberg et al., 2008; Dirks et al., 2016). Social support could be seen as a stress-reducing resource (Jean et al., 2018). Mothers and fathers with the highest psychological stress expressed a high need for support in terms of information about their child’s deafness, their functioning, and access to groups of parents in similar situations (Spahn et al., 2001). With regard to social support, early studies focused on mothers and showed that when they are more stressed, they are less satisfied with their social support (Quittner et al., 1990; Lederberg and Goldbach, 2002). Specifically, when comparing mothers of DHH children with mothers of children with normal hearing, the former reported smaller social networks, less frequent contact with family and friends, and overall less social support. They also frequently named health care professionals as a major source of support. However, mothers of DHH children seem to be as satisfied with their social support as mothers of children with normal hearing (Quittner et al., 1990; Meadow-Orlans, 1994). Fathers of DHH children reported a greater degree of support from their environment than fathers of children with normal hearing (Mavrogianni and Lampropoulou, 2020). Comparing mothers and fathers, Zaidman-Zait et al. (2017) found that mothers perceived the same amount of support as fathers, while Hintermair (2006) found that mothers perceived less support from friends, spouse, other parents of DHH children, and professionals than fathers.

Social support is a broad concept that nevertheless includes two more specific notions: formal and informal support. Formal support is support from care professionals and institutional services such as early intervention centers and social services, while informal support is that provided by personal relations such as spouse, family, and friends (Rodrigo et al., 2007). Formal support can be information received about the child’s health. In McNeil and Chabassol (1981) had already observed that mothers most often received this information from professionals, while fathers received it from mothers. Despite changing parenting roles and practices and increasing gender equality in France, Muñoz et al. (2015) made the same observation. In the education of a DHH child, few surveys have investigated the distinction between these two types of support (Poon and Zaidman-Zait, 2014), we only know that mothers with more formal support are less stressed (Zaidman-Zait et al., 2016). Behind the correlation between parenting stress and social support, the concept of social support has its own value when it comes to supporting parents of DHH children (Poon and Zaidman-Zait, 2014). Evaluating the needs for formal and informal social support could lead to a better understanding of their issues and improve prevention and intervention activities within family-centered services (Bailey and Simeonsson, 1988).

The first objective of this study was to assess parenting stress of mothers and fathers of DHH children relative to the norm and to compare their stress levels in response to both child and parental characteristics. The second objective was to assess social support needs and to see if a particular stress profile influenced these needs.

## 2. Materials and methods

### 2.1. Participants

Initially, 48 parental couples were recruited but results of 21 couples (43.75%) were discarded because at least one parent in the couple did not complete all the questionnaires in the survey, despite reminders from our two recruiting psychologists. Nine couples were excluded because the father did not complete his participation, 1 because the mother did not complete her participation and 2 because both parents did not complete their participation. We also discarded 4 couples because they did not meet the inclusion criteria. In addition, when the Parenting Stress Index score was invalid, for 1 mother and 4 fathers, their parental couples had to be excluded from statistical processing. Our sample was finally composed of 27 French parental couples of DHH children. Their demographic characteristics are described in Table 1. It should be noted that the children of non-participating parental couples are statistically older than the children of participating parental couples. These children were on average 6 years old (*U* = 62, *p* = 0.003). The other demographic characteristic variables (e.g., parents’ level of education, children’s gender, degree of deafness), show no statistical difference. Our sample is composed of DHH children, born between 2012 and 2021, presented with bilateral, moderate to profound neonatal hearing loss, diagnosed further to an uncertain early neonatal hearing screening. A genetic cause has been confirmed by the presence of pathogenic variants in a gene known to be implicated in non-syndromic genetic deafness. Non-syndromic deafness means that the children have no associated disabilities or developmental delay. All participants are hearing parents and are fluent French speakers and all children learn and use oral language as their first language. Only one parental couple in our sample was no longer in a conjugal relationship, all the others had been together for 11.31 years on average (*SD* = 4.19). Given these socio-demographic characteristics, our sample cannot be considered representative of the entire population of French parents of DHH children.

**Table 1:** Socio-demographic data of the population.

	Mothers (*N* = 27)	Fathers (*N* = 27)
Age, *M* (*SD*)	36.41 (4.37)	37.59 (4.53)
Level of education, *N* (*%*)		
Baccalauréat or less	4 (14.81)	4 (14.81)
Bachelor’s degree or equivalent	12 (44.44)	10 (37.04)
Master’s degree or PhD	11 (40.74)	13 (48.15)
French sign language or cued speech use, *n* (*%*)		
	16 (59.26)	13 (48.15)
	Family characteristics	
Children		
Female, *N* (*%*)	14 (51.85)	
Age, *M* (*SD*)	3.48 (1.99)	
Degree of deafness, *N* (*%*)		
Moderate	9 (33.33)	
Severe	6 (22.22)	
Profound	12 (44.44)	
Progressive deafness*, *N* (%)	5 (18.52)	
Size of city of residence*, N* (%)		
<2,000 persons	5 (18.52)	
2,000–5,000 persons	6 (22.22)	
5,000–20,000 persons	8 (29.63)	
20,000–50,000 persons	3 (11.11)	
>50,000 persons	5 (18.52)	
Distance from care center, *N (%)*		
0–10 km	7 (25.93)	
10–20 km	5 (18.52)	
20–50 km	7 (25.93)	
More than 50 km	8 (29.63)	

Children range in age from 1.5 to 8 years old.

*Progressive deafness involves a gradual loss of the threshold of auditory perception.

### 2.2. Procedure

The study protocol was approved by the authors’ university research ethic committee (reference number 1/2021). Parents were recruited between July 2021 and October 2022 and came from several French cities, thanks to a collaboration between the Rare Disease Reference Center for Genetic Deafness, at Lille University Hospital and other French genetic department and hospital centers. Geneticists selected families with the inclusion criteria described above through a local database. Two psychologists from this center were in charge of recruitment, contacting parents who matched inclusion criteria and explaining the survey to them. Parental couples who were interested in participating signed the consent charter, then they were contacted by phone or videoconference and orally answered a background questionnaire. Later they received a first online questionnaire, filled it in, and then received the second one. We randomized both questionnaires, which included Likert-type scales. The two questionnaires described below were completed by participants on two different platforms: the Parenting Stress Index was completed on the IRPCANADA publisher’s platform and the Family Need Survey on the university’s secure internal platform (LimeSurvey). Mothers and fathers were asked to complete questionnaires independently.

### 2.3. Measures

#### 2.3.1. Parenting stress

The Parenting Stress index (PSI) is a questionnaire that assesses different sources of parenting stress (Abidin, 1990). The French version of PSI-4, translated and validated by Bigras et al. (1996) was used. The PSI consists of a series of 101 items and 19 propositions related to life stress. The 101 items allow a total stress score to be measured in relation to two stress domains: the stress related to child characteristics (Child Domain) and the stress related to parental characteristics (Parent Domain). The Child Domain includes 47 items. These items are divided into six sub-domains. Hyperactivity (9 items) which measures the child’s behavioral expressions that may indicate attention deficit/hyperactivity. Adaptability (11 items) which measures the child’s ability to adapt to changes in the environment. Reinforcement (6 items) which shows the extent to which the parent is positively reinforced by the child in their interactions. Demands (9 items) which measures the parent’s ability to demand things of the child. Mood (5 items) which measures the child’s emotional state. Acceptability (7 items) which measures whether the child’s characteristics correspond to the parent’s expectations. Hyperactivity, Adaptability, Mood and Demands relate to the child’s temperament, whereas Reinforcement and Acceptability relate to the parent’s expectations of their child and the feeling of being rewarded by the child. The Parent Domain includes 54 items, which are divided into seven sub-domains: Competence (13 items), which measures whether the parent is able and comfortable performing the role; Social Isolation (6 items), which measures the social support available to the parent; Attachment (7 items) which measures the parent’s ability to observe and respond to the child’s needs appropriately as well as the intimacy that the parent has with the child; Parent Health (5 items), which measures how the parent’s health may contribute to their stress; Role Restriction (7 items), which measures the parent’s ability to restrict their freedoms and personal identity in the course of parenting; Depression (9 items), which measures the emotional state of the parent; Marital Relationship (7 items) which measures whether the parent feels emotionally and physically supported by the second parent. Competence and Depression are interrelated and influence the parent’s Attachment to their child. Isolation, Parent Health, Role Restriction and Marital Relationship include various situational variables other than the parent–child relationship, which can accentuate the level of stress experienced on a daily basis and thus affect the parent–child relationship. Total Stress measures the parent’s overall experience of stress, risk of dysfunction, and child’s behavioral problems. Parents are asked to express whether they “Strongly Agree,” “Agree,” are “Not Sure,” “Disagree,” or “Strongly Disagree” with the various items offered. The duration of the test is estimated between 20 and 25 min, although no restrictions are imposed. Cronbach’s alpha coefficient ranged from 0.78 to 0.88 in the Child Domain, from 0.75 to 0.87 in the Parent Domain and is 0.98 for Total Stress.

#### 2.3.2. Parental needs of social support

The Family Need Survey (FNS) is a questionnaire assessing needs of parents of a child with disabilities. Created by Bailey and Simeonsson (1988), revised and validated in Bailey et al. (1992), it has been used for research on parental needs in several geographic and clinical contexts, including parents of DHH children, where its usefulness in improving multidisciplinary care of deaf children has been demonstrated (Dalzell et al., 2007). We used its translated French version (Dell'Armi, 2016). FNS consists of 25 items divided into 7 categories: “Information Needs,” “Family and Social Support Needs,” “Financial Needs,” “Explaining to Others,” “Child Care,” “Professional Support,” and “Community Services.” Parents choose between 3 responses for each item (“I definitely do not need help with this,” “I’m not sure,” “I definitely need help with this”), which we score, respectively, 0, 1, and 2. Cronbach’s alpha coefficient is 0.91 for total score and 0.65–0.86 in the sub-domains. The reliability of the test at 6 months ranges from 0.67 to 0.81 (Sexton et al., 1992). We created two scores of Formal and Informal Support needs, categorizing the items according to the definition of these two domains. Categorization was performed separately by the first author and by a doctoral student who was not involved in the research.

#### 2.3.3. Statistical analysis

All analyses were performed using the 2.2.5. version of Jamovi (The Jamovi Project, 2022). Shapiro–Wilk’s test of normality shows that the majority of dependent variables do not follow a normal distribution, in addition, considering that parental couples by definition include non-independence of data due to the existence of a well identified dyad (Kenny et al., 2020), non-parametric tests suited for related samples were chosen. Specifically, to compare PSI scores of mothers and fathers to the standardized test norm and for between-group comparisons (i.e., to compare mothers’ and fathers’ PSI and FNS scores) Wilcoxon’s rank test was used (Woolson, 2007). To test the homogeneity of variances, the Levene test was used. All *p*-values reported were from two-tailed tests and were under 0.05 to be considered significant.

## 3. Results

### 3.1. Parenting stress

Levene’s test revealed a difference between the variances of mothers’ and fathers’ PSI scores, except for the Life Stress sub-domain (*p* < 0.01). Indeed, fathers have less distributed scores than mothers.

As shown in Table 2, in our population, being a mother or a father of a DHH child is not associated with higher Total Stress scores on PSI, neither in the Child Domain nor in the Parental Domain. However, in both Child Domain and Parental Domain, some sub-domain scores of mothers or fathers are different from the norm. In the Child Domain, the Hyperactivity sub-domain score shows a higher level of stress in mothers (*z* = 222.5, *p* = 0.039) and in fathers (*z* = 257.5, *p* = 0.011). Only fathers show a significantly higher level of stress related to the Adaptability sub-domain score (*z* = 190.5, *p* = 0.009). Mothers (*z* = 20, *p* < 0.001) and fathers (*z* = 29, *p* < 0.001) have lower stress in the Reinforcement sub-domain score and only mothers have significant lower stress in the Acceptability sub-domain score (*z* = 70, *p* = 0.013). In the Parent Domain, mothers have lower stress levels related to the Attachment sub-domain score (*z* = 61, *p* = 0.006) but they show higher stress levels in both Parent Health sub-domain score (*z* = 273, *p* = 0.045) and Role Restriction sub-domain score (*z* = 320, *p* = 0.002), fathers have lower levels of stress in the Marital Relationship sub-domain score (*z* = 54.5, *p* = 0.004). Both mothers (*z* = 83.5, *p* = 0.011) and fathers (*z* = 58.5, *p* = 0.003) have lower stress levels related to the Life Stress sub-domain score.

**Table 2:** Comparison of mothers’ and fathers’ PSI T-scores to the norm.

	Mothers		Fathers	
	Mean (SD)	Median (IQR)	Mean (SD)	Median (IQR)
Total stress	51.7 (8.32)	51 (45–56)	50.4 (2.90)	50 (49–51)
Life stress	46.1 (7)	45 (41–49.5)*	46.2 (5.52)	45 (42.5–47.5)**
Child domain	51.1 (9.54)	47 (45.5–55.5)	50.6 (3.47)	50 (48–51.5)
Hyperactivity	53.6 (8.19)	53 (49–56.5)*	52.1 (3.77)	52 (49.5–55)*
Adaptability	54.3 (11.21)	53 (47.5–59.5)	52.1 (4.25)	51 (50–53)**
Reinforcement	42.8 (6.31)	42 (38–47)***	47.2 (3.03)	46 (45–49)***
Demands	53.5 (11.62)	51 (45–59)	50.9 (3.79)	51 (48.5–53)
Mood	53.8 (11.22)	53 (45–60.5)	50.8 (4.22)	51 (49–52)
Acceptability	45.7 (9.02)	43 (39–50)*	48.5 (4.27)	48 (45–51.5)
Parent domain	52.1 (8.54)	55 (45–57.5)	50 (2.41)	50 (49–50.5)
Competence	51 (9.07)	52 (44–57)	49.1 (3.42)	49 (47–50)
Social isolation	51.4 (10.18)	52 (44–59.5)	51 (3.56)	51 (49–52.5)
Attachment	45.7 (6.18)	45 (41–49)**	50.8 (3.27)	51 (49–52.5)
Parent health	55.2 (10.46)	52 (47–64)*	51.6 (6.73)	51 (47–52)
Role restriction	58.3 (10.16)	59 (49–67)**	51.2 (3.31)	51 (49.5–52.5)
Depression	52.4 (9.68)	52 (45–59)	50 (3.28)	49 (48–51)
Marital relationship	49 (9.1)	51 (43–56.5)	48.2 (3)	48 (47–49.5)**

***p < 0.001, **p < 0.01, *p < 0.05.

In Table 2, when comparing mothers’ and fathers’ PSI scores we can only see differences in the sub-domain scores of Acceptability (*z* = 100, *p* = 0.033), Reinforcement (*z* = 67.5, *p* = 0.004), Attachment (*z* = 59, *p* = 0.002), and Role Restriction (*z* = 308, *p* = 0.004). Mothers have higher stress scores in Role Restriction sub-domain than fathers. Fathers show higher stress levels than mothers in the sub-domains Acceptability, Reinforcement and Attachment.

### 3.2. Needs for social support

Table 3 shows raw and weighted FNS scores for mothers and fathers. We first searched for any differences in needs between mothers and fathers, Wilcoxon’s rank test shows no difference. We compared the need for formal and informal support among mothers and among fathers, weighting both scores by the number of items included. Wilcoxon’s rank test shows no difference between the need for Formal and Informal Support among mothers but among fathers the Formal Support need is significantly higher than the Informal Support need (*z* = 269, *p* = 0.004).

**Table 3:** Raw and weighted FNS scores of mothers and fathers.

	Raw scores				Weighted scores*			
	Mothers		Fathers		Mothers		Fathers	
	Mean (SD)	Median (IQR)	Mean (SD)	Median (IQR)	Mean (SD)	Median (IQR)	Mean (SD)	Median (IQR)
Total score	27.48 (12.24)	26 (20–34.5)	23.41 (16.43)	21 (9–33.5)	0.79 (0.35)	0.74 (0.57–0.99)	0.67 (0.47)	0.60 (0.26–0.96)
Formal support	19.9 (9.61)	19 (15–26)	18.1 (12)	17 (8–26)	0.83 (0.4)	0.79 (0.63–1.08)	0.76 (0.5)	0.71 (0.33–1.08)
Informal support	7.44 (4.7)	8 (4–11.5)	5 (5.05)	4 (1–8)	0.83 (0.52)	0.89 (0.44–1.27)	0.55 (0.56)	0.44 (0.11–0.89)
Information needs	8.15 (3.01)	8 (6–10)	7.41 (3.63)	7 (5.5–9)	1.16 (0.43)	1.14 (0.86–1.43)	1.06 (0.52)	1 (0.78–1.29)
Family and social support needs	6.07 (4.25)	7 (2–9.5)	3.96 (4.54)	2 (0.5–6)	0.76 (0.53)	0.88 (0.25–1.19)	0.5 (0.57)	0.25 (0.06–0.75)
Financial needs	4.19 (3.56)	3 (0.5–7.5)	3.96 (3.72)	3 (0–6)	0.7 (0.59)	0.5 (0.08–1.25)	0.66 (0.62)	0.5 (0–1)
Explaining to others	3.19 (2.62)	3 (1–5.5)	2.59 (2.86)	1 (0.5–4.5)	0.64 (0.52)	0.6 (0.2–1.1)	0.52 (0.57)	0.2 (0.1–0.9)
Childcare	1.37 (1.42)	1 (0–2)	1.48 (1.5)	1 (0–3)	0.46 (0.47)	0.33 (0–0.67)	0.49 (0.5)	0.33 (0–1)
Professional support	1.78 (1.53)	2 (1–2.5)	1.59 (1.39)	2 (0–2)	0.59 (0.51)	0.67 (0.33–0.83)	0.53 (0.46)	0.67 (0–0.67)
Community services	2.74 (1.91)	3 (1–4)	2.41 (2.1)	2 (0.5–4)	0.91 (0.64)	1 (0.33–1.33)	0.8 (0.7)	0.67 (0.16–1.33)

*The raw scores were weighted according to the number of items in each category.

**Figure 1:**
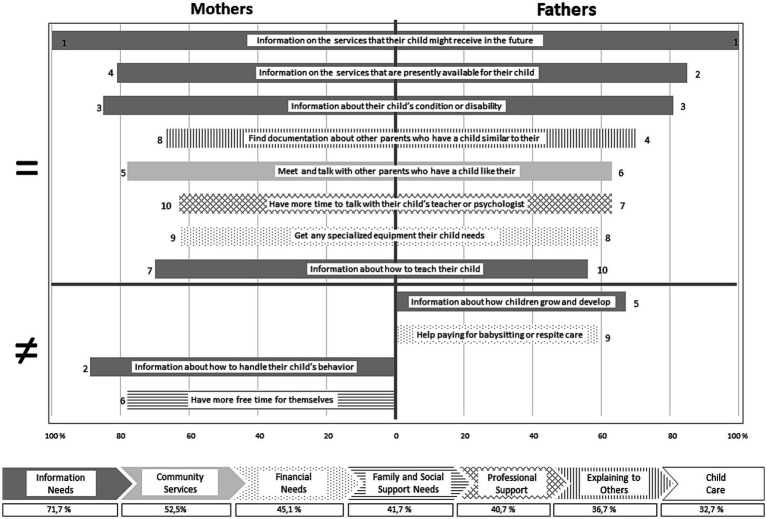
Top ten social support needs of mothers and fathers of DHH children.

All of these social support needs items are included in the seven FNS categories and among these categories, and considering all the items, the levels of need expressed by parents differs. These levels are given by the average of the percentages of parents who expressed a need for each item in the category as shown in Figure 1, thus in decreasing order of importance: 71.1% of parents have information needs, 52.5% have community service needs, 45.1% have financial needs, 41.7% have family and social support needs, 40.7% have professional support needs, 36.7% have ‘explaining to others’ needs and 32.7% have child care needs.

For a more accurate understanding of parents’ social support needs, the percentage of fathers and mothers who rated a need for support was calculated for each item, extracting the top 10 needs. These needs are also shown in Figure 1. Thus, we observed that eight of the top 10 needs expressed by parents are similar for mothers and fathers. Hundred percent of our participants expressed the need for information about the services that their child might receive in the future, which is the first and foremost need. Then, even if seven items are similar, the classification differs. The need for information about the services that are presently available for their child is the second need for fathers (85%) and fourth for mothers (81%). The need for information about their child’s condition or disability is the third need for both fathers (81%) and mothers (85%). The need to find documentation about other parents who have a child similar to theirs is the fourth need for fathers (70%) and the eighth for mothers (67%). The need to meet and talk with other parents who have a child like theirs is the sixth need for fathers (63%) and the fifth for mothers (78%). The need to have more time to talk with their child’s teacher or psychologist is the seventh need for fathers (63%) and tenth for mothers (63%). The need to get any specialized equipment their child needs ranks eighth for fathers (59%) and ninth for mothers (63%). The need for information about how to teach their child is tenth for fathers (56%) and seventh for mothers (70%). Some of the top 10 expressed needs differ for mothers and fathers. Thus, the fifth need for fathers is that of information about how children grow and develop (67%) and their ninth need is for help paying for babysitting or respite care (59%). The second need for mothers is that of information about how to handle their child’s behavior (89%) and their sixth need is to have more time for themselves (78%).

### 3.3. Link between parenting stress levels and needs for social supports

**Figure 2:**
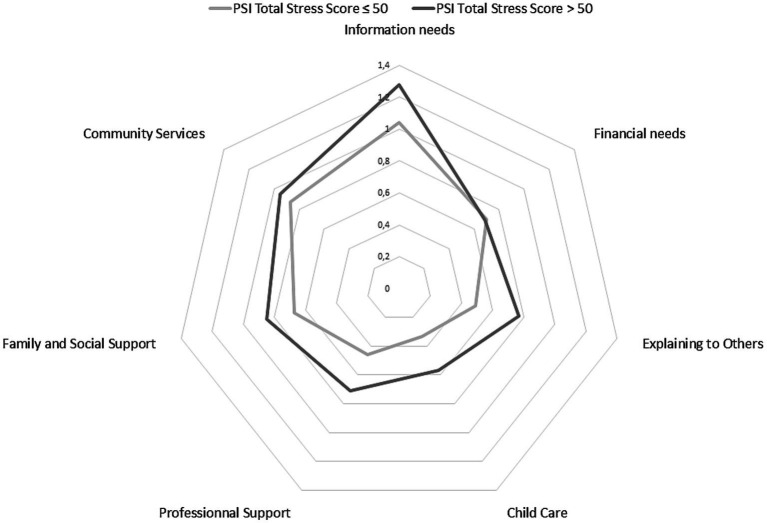
Profile of social support needs of mothers of DHH children.

**Figure 3:**
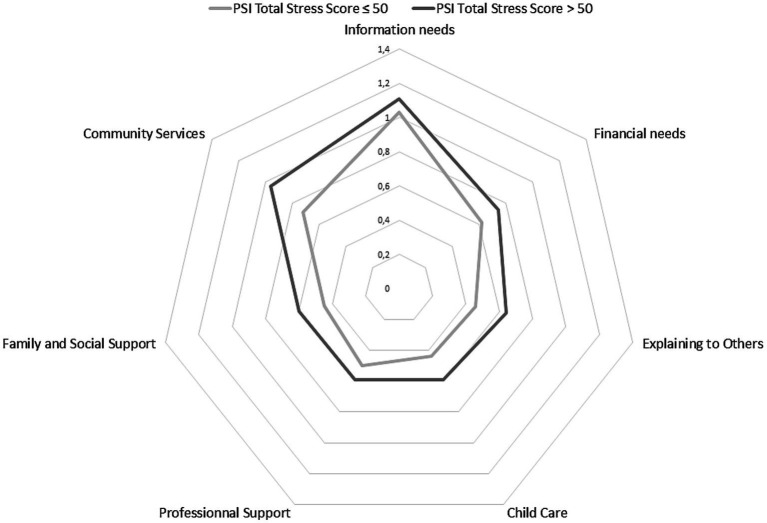
Profile of social support needs of fathers of DHH children.

To highlight the links between parenting stress and social support needs, we differentiated parents’ social support needs profiles according to stress levels. We observed that the profiles of different categories of social support needs varied according to parents’ stress levels, so that parents with stress level over and above the norm had higher social support needs profiles than parents with stress levels at or below the norm. Differences between mothers and fathers of DHH children appear and are presented in Figures 2, 3.

Figure 2 shows that mothers who register stress levels over and above the norm have a higher overall need for social support than mothers who are less stressed, except when it comes to financial needs.

Figure 3 shows that fathers who register stress levels over and above the norm have a higher overall social support need than fathers who are less stressed.

## 4. Discussion

Although it cannot resolve the lack of consensus on parenting stress in parents of DHH children, the present study attempts to contribute, particularly concerning the potential differences between mothers and fathers of DHH children. As Cryer-Coupet and Gibson (2022) point out, it is difficult to recruit fathers for research; this was indeed the case in our study, where 75% of parental couples who did not follow through with their participation were excluded because of fathers. We had the opportunity to include parents living in couples, which is an original feature of our study, but also a limitation to the generalization of our results to parents who would otherwise be isolated. As is often the case, due to field constraints, it was conducted with participants of a fairly high socioeconomic level, although this is known to protect from parenting stress (Brand and Coetzer, 1994). The parental couples who participated had younger children than the parental couples who did not, suggesting that it is relevant in this type of study of parenting experience to include parents early in the diagnosis process. Moreover, the children were a special group in that they had been screened and diagnosed early. It provides knowledge about parents of DHH children in France since this study was the first to be conducted in France where the systematization of early hearing screening only appeared in 2012.

### 4.1. Parenting stress

The results of our research do not suggest a higher level of parenting stress in parents of DHH children than in parents of children with normal hearing. This corroborates the results of previous studies that have not shown an increase in parenting stress when the child is DHH (Lederberg and Goldbach, 2002; Åsberg et al., 2008; Chang et al., 2022). However, the PSI that assesses the level of parenting stress is often used in its short form and very rarely in its full form as in our study. Only Meadow-Orlans (1995) and Meadow-Orlans et al. (2004), have used the full PSI in a study providing the same level of precision as ours in terms of observed and commented sub-domains. Indeed, if the scores obtained in the child and parental domains do not differ between hearing parents of DHH children and parents of children with normal hearing, a number of differences can nevertheless be noted. In the child domain, our results confirm previous research by Meadow-Orlans (1995) and Meadow-Orlans et al. (2004), that mothers and fathers perceive their child as more hyperactive than parents of children with normal hearing. Although the PSI is not specifically designed for parents of DHH children, as a measure of parenting stress it does offer an interpretation for each sub-domain when scores are above the norm (Abidin, 1990). For instance, the fact that parents of DHH children are more stressed by their child’s hyperactivity could be explained by the fact that these are children who require more energy to raise. However, our results differ with respect to adaptability, reinforcement, and acceptability. In our study, fathers view their child as less adaptable and more difficult to raise because of their greater difficulty in adapting to changes in the physical and social environment. Comparing to parents of children with normal hearing, mothers and fathers of DHH children are less stressed by reinforcement, and they perceive their child as a greater source of gratification, which gives them a positive self-image. In our sample, the perception of the child as a source of gratification is even more prevalent among mothers than among fathers. Finally, mothers of DHH children are less stressed in the sub-domain of acceptability, compared with mothers of normal hearing children and with fathers of DHH children. In research on prematurity, Ruiz et al. (2020) show that fathers focus on their parenting role while mothers focus on the child’s needs and feelings, and are therefore less focused on their own role, making acceptance of the child easier. Although no studies have been conducted on this subject in the field of deaf studies, it can be assumed that the same mechanisms are present in these parents of DHH children. In addition, we did not find that fathers of DHH children are more stressed than fathers of children with normal hearing in the sub-domains of acceptability and demands. These discrepancies may be explained by the age of the children at the time of PSI with parents in the two studies. Whereas Meadow-Orlans (1995) and Meadow-Orlans et al. (2004) interviewed parents of 9-month-old DHH children, we interviewed parents of older children. The latter have more experience with their child and the management of language and hearing rehabilitation is more advanced. Moreover, even in the case of cochlear implants, time allows a regularization of the stress level (Spahn et al., 2003). In the parental domain, our results confirm Meadow-Orlans (1995) and Meadow-Orlans et al. (2004) study that mothers and fathers of DHH children differ in the sub-domain of role restriction, with mothers feeling that parenting has a worse impact on their sense of freedom. We add that these mothers are also more stressed in this sub-domain than parents of children with normal hearing. As in Meadow-Orlans (1995) study, fathers show higher scores than mothers in the Attachment sub-domain, indicating that they have difficulty feeling an emotional connection to their child. Surprisingly, although studies on attachment are widespread in developmental psychology, none of these studies address the issue of attachment quality for hearing fathers with their DHH children (Szarkowski and Dirks, 2021). We add that mothers of DHH children are less stressed in the Attachment subdomain compared to parents of children with normal hearing, indicating a deeper emotional bond with their child. Finally, in our study, fathers rate their marital relationship more positively, indicating stronger support from their spouse, whereas mothers do not. We also found that mothers are less healthy, which may be due to their parental responsibilities (Abidin, 1990).

The PSI is a widely used test to measure parenting stress, but it only assesses general parenting stress, i.e., it does not take into account specific stressors due to the context of childhood hearing loss and its particular demands (Quittner et al., 2010; Zaidman-Zait et al., 2016). In fact, Quittner et al., (2010) that parents of DHH children, although they do not have higher levels of general parenting stress, do have higher levels of context-specific stress.

Unexpectedly, we found less dispersion of PSI scores among fathers than mothers, with fathers’ scores actually being more clustered. This may be related to the fact that fathers tend to underestimate their stress and anxiety in order to protect their spouses or themselves and thus their stress profiles are more normalized (Baldoni et al., 2021; Filippa et al., 2021; Stefana et al., 2022). It is also important to consider that most tests used in developmental psychology, although offered to both mothers and fathers, are designed within a theorical and cultural context that focuses on what science knows about mothers’ functioning, whereas fathers tend to conceive their difficulties differently from mothers.

### 4.2. Needs for social support

FNS scores show no difference between mothers and fathers in terms of formal and informal support needs. When comparing formal and informal support needs in mothers no difference is apparent, while fathers express more needs for formal support than informal support. This result is consistent with Hintermair and Sarimski (2019) findings that fathers have a strong need for social support from care professionals. This may be related to the fact that it seems easier for men to express and expect help from professional care services, but also to the fact that fathers receive less formal support, because, for example, information about the child’s situation is passed on by professionals to mothers, whereas fathers have to rely on mothers for this information (McNeil and Chabassol, 1981). This may also be related to a potential under-expression of informal support needs, which could be due to the cultural context that traditionally stigmatizes men’s emotions and prevents them from seeking this form of support (Vogel et al., 2014).

The FNS provides a clear list of the most important social support needs. Mainly parents need to have information in particular about future and current services for their child, as well as information about the child’s condition, deafness, and the specificity of learning in this particular context and how to teach their child things. This need has already been identified by several studies (e.g., Spahn et al., 2001; Muñoz et al., 2015), Luterman and Kurtzer-White (1999) specified that they needed unbiased and complete information. As with the study of Fitzpatrick et al. (2008), ours shows that parents need information on how to find documentation on other parents who have a child similar to their own. Care services should encourage the creation of spaces for parents to meet and talk, thus meeting one of their priority needs. Parents of DHH children also express the need to have more time to exchange with their child’s specialists and to find professionals who are both specialized in deafness and attentive, this could be linked to a need for the coordination of services (Fitzpatrick et al., 2008). Financially, they need help financing specialized equipment, although significant provision is already available in France. In our study, differences appear between some needs of fathers and mothers of DHH children: in fact, fathers primarily need information about their child’s development, while mothers need information about managing their child’s behavior. Mothers express the need for time off, while fathers express the need for financial support for respite care. Researchers and clinicians should focus on distinguishing the specific needs of fathers and mothers and tailoring support accordingly. The percentages of parental needs by category show us that despite the existing support, there remains a significant lack of social support, hence the need for a family-centered approach to service delivery, beyond early intervention and therefore throughout the child’s development (Jamieson et al., 2011).

Overall, we encourage clinicians to consider these guidelines for improving support for parents of DHH children, but as recommended by Dalzell et al. (2007), we also encourage them to use the FNS as a personalized tool to assess the needs of each of their patients’ parents, allowing them to enable the most appropriate supports for each.

The stress profiles of mothers and fathers of DHH children lead us to believe that when these parents have higher than normal stress levels, they tend to express greater social support needs than parents with normal or subnormal stress profiles. These profiles lead us to consider a correlation between parenting stress and social support needs, so our results seem to follow research that has shown such a correlation between these two factors (e.g., Dirks et al., 2016).

## 5. Conclusion and implications

The results of this study show us that while, in general, mothers and fathers may have relatively similar levels of parenting stress, there are finer differences in the expression of this stress and the characteristics of the child or parent that influence them, as well as in the expression of their social support needs.

Mothers and fathers of a parental couple cannot be considered as non-dependent, dyadic statistical analysis allows mothers-fathers influences to be considered. Unfortunately, due to the limited sample size of the present study, it was impossible to perform a more complete and detailed dyadic analysis (Kenny et al., 2020). In line with the recommendations of Szarkowski and Dirks (2021), this study includes fathers and not only mothers. Although some studies are beginning to focus on fathers, they are still under-represented, and their specifics are poorly understood. This research brings an additional stone to the building of knowledge and understanding of parenting stress and social support needs of mothers and fathers of DHH children. Nevertheless, other concepts should be included in future research, especially in France where the lack of studies is glaring. Indeed, parenting stress is influenced by coping resources (Hintermair, 2006; Gurbuz et al., 2013), self-efficacy (Zaidman-Zait et al., 2016, 2017), and involvement, which in turn influences self-efficacy (Ingber and Most, 2012; Hintermair and Sarimski, 2019) and social support (Dirks and Szarkowski, 2022). Currently, fathers and mothers of DHH children are sinking into the traditional division of roles: mothers providing daily care and participation in early medical interventions for children, and fathers working more to support the family and meet the financial demands of the cost of deafness. Furthermore, fathers are still not included in care services by mothers and professionals, even though their involvement is crucial for them and for the child (Hintermair and Sarimski, 2019). Consistently with our study, where we emphasize the primary interest in studying parenting stress and social support needs of parents of DHH children, the fact is that levels of parenting stress have been associated with the poor functioning and development of DHH children (e.g., Pipp-Siegel et al., 2002; Hintermair, 2006). Future studies on these factors need to be conducted to improve early intervention programs for parents of DHH children, both for themselves and for their child.

## Data availability statement

The raw data supporting the conclusions of this article will be made available by the authors, without undue reservation.

## Ethics statement

The studies involving humans were approved by the Université de Picardie Jules Verne ethic committee (reference number 1/2021). The studies were conducted in accordance with the local legislation and institutional requirements. The participants provided their written informed consent to participate in this study.

## Author contributions

AM realized the statistical analysis and drafted the manuscript. LC, MC, CV, and CV-D conducted the data collection. BLD conceived, coordinated the study, and revised the manuscript. All authors approved the final manuscript.

## Funding

This research was supported by the *Fondation Maladies Rares* and *AG2R La Mondiale* (U20201203).

## Conflict of interest

The authors declare that the research was conducted in the absence of any commercial or financial relationships that could be construed as a potential conflict of interest.

## Publisher’s note

All claims expressed in this article are solely those of the authors and do not necessarily represent those of their affiliated organizations, or those of the publisher, the editors and the reviewers. Any product that may be evaluated in this article, or claim that may be made by its manufacturer, is not guaranteed or endorsed by the publisher.
